# VGLM proportional odds model to infer hosts’ Airbnb performance

**DOI:** 10.1007/s11135-022-01550-2

**Published:** 2022-10-20

**Authors:** Giulia Contu, Luca Frigau, Marco Ortu

**Affiliations:** Department of Economics and Business Sciences, University of Cagliari, Viale Sant’Ignazio 17, 09123 Cagliari, Italy

**Keywords:** Occupancy rate, Overall rating, Airbnb, Host performance, Proportional odds model

## Abstract

We investigated aspects of host activities that influence and enhance host performance in an effort to achieve best results in terms of the occupancy rate and the overall rating. The occupancy rate measures the percentage of reserved days with respect to available days. The overall rating identifies the satisfaction level of guests that booked an Airbnb accommodation. We used the proportional odds model to estimate the impact of the managerial variables and the characteristics of the accommodation on host performance. Five different levels of the occupancy and the overall rating were investigated to understand which features impact them and support the effort to move from the lowest to the highest level. The analysis was carried out for Italy’s most visited cities: Rome, Milan, Venice, and Florence. We focused on the year 2016. Moreover, we investigated different impact levels in terms of the overall rating during the COVID-19 pandemic to evaluate possible differences. Our findings show the relevance of some variables, such as the number of reviews, services, and typology of the rented accommodation. Moreover, the results show differences among cities and in time for the relevant impact of the COVID-19 pandemic.

## Introduction

New forms of hospitality have arisen in the last decades. Apartments, couches, private and shared rooms, and boats are new forms of tourist accommodations. They can be booked directly on online platforms. One of the most famous online platforms for this purpose is Airbnb. It was founded in 2008 in San Francisco and has grown significantly over the years. Currently, Airbnb has over 7 million listings in 100,000 cities and 220 countries and regions (https://news.airbnb.com/about-us/) (accessed on December 9th 2021).

Numerous researchers have considered the Airbnb accommodations as an integral part of the sharing economy (Gutt and Herrmann 2015; Gutierrez et al. 2016; Quattrone et al. 2016; Lutz and Newlands 2018; Gibbs et al. 2018; Roelofsen and Minca 2018; Ert et al. 2016; Pezenka et al. 2017; Dann et al. 2019), which can be defined as *the peer-to-peer-based activity of obtaining, giving, or sharing access to goods and services, coordinated through community-based online services* (Hamari et al. 2016, p. 2048). Researchers believe that the new forms of hospitality are based on three specific elements: social and cultural exchange, personal development of people, and knowledge of more places. However, this point of view has been recently criticized, especially for accommodations rented on the Airbnb platform. For instance, Oskam and Boswijk (2016) and Ikkala and Lampinen (2015) have recognized a monetary nature of the Airbnb exchange among hosts and guests. Oskam (2019) has demonstrated the same nature of relationship between hosts and Airbnb, since the company keeps for itself a percentage of earnings by hosts (Oskam et al. 2018; Oskam 2019). In fact, some researchers (Oskam and Boswijk 2016; Ikkala and Lampinen 2015; Oskam et al. 2018; Oskam 2019; Li et al. 2015; Ke 2017a, b; Mauri et al. 2018) have offered a new point of view of the Airbnb activity, defining it as a business based on a monetary exchange.

The two different points of view generate a particularly interesting debate, which impacts on the nature of Airbnb and on the role played by hosts. From the sharing economy point of view, a host can be considered as one of the authors of an exchange finalized to share a space and increase social and cultural knowledge. On the contrary, in the approach based on a monetary exchange, the host can be considered as a manager who rents a proper space. If Airbnb is considered a business activity, then hosts should manage their activities to identify potential guests, offer high-quality services, promote their business, and, especially, earn as much money as possible. Consequently, a host can be considered a tourism worker. Hosts have been identified with the words “professional” (Li et al. 2015), *commercial* and *multi-listers* (Ke 2017a, b), and *micro-entrepreneurs* (Mauri et al. 2018). These attributes focus attention on the business nature of hosts and make it possible to consider hosts as tourism professionals. The attribution of this business nature determines the necessity of analyzing and evaluating a host activity, like other tourism activities related to an accommodation.

In the literature, few articles have focused on the study of host activities in themselves, and of the elements that can influence host performance. To overcome this gap, we focus, in this paper, on host performance to identify aspects that can enhance guest satisfaction and, consequently, improve the number of reservations. To reach these aims, we have investigated elements that can impact host performance, expressed through the occupancy rate (OcR) and the overall rating (OvR). The first element defines the capacity of a host to constantly and totally book their accommodation. The second identifies the satisfaction level of a guest that booked an Airbnb accommodation. We used, among others, the proportional odds model as defined in Yee (2015) to analyze and determine which elements can directly impact different levels of the OcR and the OvR.

Moreover, we tried to understand if a unique model can explain host activities in different tourist cities. The aim was to investigate the existence of differences that can condition host activities. The analysis focused on data related to some Italian cities. Specifically, we analyzed the most touristic ones, i.e. those with the highest number of official tourists. They are Rome, Milan, Venice, and Florence. Taking into account more cities made it possible to analyze host activities in specific areas and, at the same time, identify possible differences between host activities in these areas. Moreover, it made it possible to evaluate, if it exists, a specific model for each specific destination.

The analysis focused on 2016. Additionally, we replicated the model on data related to 2021 only for the response variable OvR. We would have conducted the analysis also for the OcR response variable but the relevant data was not available. The investigation of the OvR aimed to understand if the COVID-19 pandemic impacted elements that affect the general judgment expressed by guests. Since the pandemic significantly impacted the tourism sector and the Airbnb market (Jang and Kim 2022; Chuah et al. 2022; Jang and Kim 2022; Garha and Azevedo 2022), it was important to identify differences in variable impacts.

Although some researchers have investigated the determinants of the Superhost badge (see, for instance, Gunter 2018) and host performance (see, for instance, Contu et al. 2019; Contu 2022), the current study, to our knowledge, is the first to investigate elements that influence host performance, taking into account five different levels of the OcR and the OvR. The analysis carried out for each level made it possible to identify both the elements that characterized the different results and those that support the rise of a host from the lowest to the highest OcR and OvR values. Moreover, the analysis carried out for each city made it possible to identify which elements impacted a specific destination. In this way, the list of elements was defined with respect to a specific destination, identifying in greater detail elements that impacted the destination and those that did not. In fact, another innovative aspect was defined by the framework as the ability to support hosts in the identification of both the elements that influence their specific OvR and OcR values and the elements that affect the improvement of their performance levels. Our aim is to define a guide to support hosts in their continuous efforts to improve their performance. We believe this research presents further innovative aspects. Firstly, we have introduced a new variable in the analysis of the host performance: the distance to the city center. This variable is generally considered as a determinant of the Airbnb price (see, for instance, Zhang et al. 2017), but it was not used to evaluate the OvR. Secondly, we analyzed elements that influence guest judgments in two different phases—the pre-COVID-19 period and during the COVID-19 pandemic—with the aim to investigate possible differences due to the pandemic.

Five sections, besides this introduction, make up this study. The second section is a review of the literature related to Airbnb hosts. The third section is the research design, with research questions, data, and methodology described. The results and implications are explained in the fourth part. The last section focuses on concluding remarks, limitations, and future developments.

## Literature review

A few studies in the literature have investigated Airbnb host activity and the elements that influence host performance. Generally, host profiles have been analyzed taking into account aspects such as motivations to become hosts in the first place, monetary exchange, price definition, communication strategies, and elements that influence relationships between hosts and guests (see, for instance, Ikkala and Lampinen 2015; Lampinen and Cheshire 2016; Tussyadiah 2016; Jung and Lee 2017; Wang and Nicolau 2017; Ke 2017a; Neumann and Gutt 2017; Mauri et al. 2018; Benítez-Aurioles 2018; Gibbs et al. 2018; Tussyadiah and Park 2018). Moreover, major attention has been paid to the study of Superhost profiles and their activities (see, for instance, Gunter 2018).

Three Airbnb research areas are relevant to this study. The first research area is related to the identification of elements that define and support host and guest relationship, being that the rental of Airbnb accommodation is based on this relationship (Tussyadiah and Park 2018). These elements are crucial to improving host reliability and results. The elements identified in the literature are different. For instance, Zervas et al. (2015) discovers that reviews are fundamental to the Airbnb platform and host activities. The researchers state that reviews are important to build trust and *to help determine how listings are ranked in response to user queries* (Zervas et al. 2015, p. 2). Additionally, they find that ratings on Airbnb are more positive than those published on other famous platforms, such as TripAdvisor. The relevance of a positive Airbnb rating is crucial to entrepreneurial and platform success because it influences user choices.

Similar results have been obtained by Bridges and Vásquez (2018), wherein it is demonstrated that positive ratings and reviews are the goals of Airbnb hosts. This is so for two reasons. The first reason concerns the value of guests’ positive experience. The second reason has to do with the importance of minimizing negative experience due to different factors such as sociocultural norms of politeness, established trust between hosts and guests, lack of anonymity, direct removal by Airbnb of reviews that violate their guidelines.

Additionally, Zhang et al. (2018) discovers that perceived trust is affected by different elements, such as host reputation, response behavior of hosts to guest inquiries, semantic topics in self-descriptions used by hosts to introduce themselves and their activities, and positive facial expressions on profile photos. Moreover, Guttentag (2015) demonstrates how trust is based on different elements, such as reviews, price, and pictures. The importance of photos is also recognized by Ert et al. (2016). In fact, the authors note an important impact of photos on decision-making by guests. Specifically, as far as a guest tends to infer a host’s trustworthiness from the host’s photos, their choice is affected by this inference (Ert et al. 2016).

From the foregoing, it can be said that two elements are fundamental to the development of trust in the literature: photos and reviews.

The second relevant research area is related to the needs of guests and satisfying their expectations. It is important to understand the characteristics of potential clients, the motivations behind guest behaviors, and consumers’ reasons in order to define the best market (Guttentag et al. 2018) and build the best offer. Gunter and Önder (2018) discoveres that Airbnb demand is positively influenced by the variables distance from the city center, response time of hosts, and Instant Book. On the contrary, variables such as Extra People Fee and Cleaning Fee have a negative impact on the Airbnb demand. Festila and Müller (2017) argues that *factors such as good value for money, proximity to interest points, and access to residential neighborhoods incentivize individuals to consider Airbnb as a viable alternative to traditional accommodation* (Festila and Müller 2017, p. 60). Moreover, the authors highlight how Airbnb consumption is affected by the desire for meaningful, life-enriching experiences through access to characteristic and personalized accommodation. Similar results have been obtained by Oskam (2019). For Guttentag et al. (2018), there are five motivations behind an Airbnb choice, which include, in order of importance, interaction with the host and locals, home benefits, novelty, sharing economy ethos, and local authenticity. For Varma et al. (2016), the elements that affect a guest’s choice of accommodation are price, image/reputation, reviews, availability of the facilities on the web, recommendation of friends, security, service quality, staff behavior, cleaning arrangement, appearance, location, access to transportation, and past experience (Varma et al. 2016, p. 232). Finally, So et al. (2018) discovers that aspects such as unfamiliarity, sustainability, community, social interaction, and sharing economy ethos do not influence guest motivations. However, factors such as price value, enjoyment, and home benefits are relevant to the perception of Airbnb accommodations as alternatives to hotel lodgings. Additionally, enjoyment and home benefits are motivational aspects that influence an Airbnb choice.

To summarize, elements that influence the choice of an Airbnb accommodation are related to the provided services, price, image of the host, and relationship between the host and the guest.

Finally, the third research area is related to Superhosts and their performance. Airbnb attributes the Superhost badge taking into account four different aspects. Specifically, Airbnb declares that to be a Superhost it is necessary, in the previous 12 months, to have hosted at least 10 stays, honored every reservation unless there is an extenuating circumstance, responded within 24 hours at least 90% of the time, and achieved a 4.8+ overall rating (https://www.airbnb.com/Superhost?locale=en) (accessed on December 9th 2021).

Some researchers have investigated elements that impact badge attribution and Superhost activity. For instance, Gunter (2018) attempts to comprehend if the variables used by Airbnb to define the Superhost status really impact the probability of becoming a Superhost. The investigation focuses on Superhosts in San Francisco. The results show the relevant impact of the variable Overall rating and the existence of a specific ranking. In this ranking, the first position is occupied by the *Overall rating*, followed by the cancellation policy, the response rate, and the number of bookings. This work is later broadened in Contu et al. (2019), with a new group of variables included in the model. This group of variables, which has been called the managerial variables, identifies aspects that can be directly chosen by hosts, such as the minimum and maximum nights for reservations, how far in the future guests can book, and cleaning fees. The results show how the managerial variables impact the probability of becoming a Superhost.

Other researchers have investigated the Superhost profile and the impact of the badge on accommodation management. For instance, Liang et al. (2017) discovers that accommodations managed by Superhosts are more likely to receive reviews than those managed by normal hosts, and that guests prefer to spend more money on accommodations with the Superhost badge. Similar results have been obtained by Ma et al. (2017). The study finds a positive relationship between the Superhost status and the price of accommodation, demonstrating an increase in income for Superhosts. The study also discovers that Superhosts have significantly more extensive host profile. Moreover, Roelofsen and Minca (2018) underlines becoming a Superhost gives greater visibility on the Airbnb platform, and Xie and Mao (2017) highlights how being a Superhost, a longer operating experience, and a higher response rate impact reservations.

## Research design

### Research questions

This study was focused on two specific objectives. Firstly, we investigated the variables that can directly influence host performance, defined by the two variables OvR and OcR. The former describes the level of customer satisfaction with the whole Airbnb experience. It is an expression of the level of satisfaction of guests and can be considered as a measure of the quality of services offered by the host, the beauty of the accommodation, and the kindness of the host. The second variable is a measure that is normally used to evaluate hotel performances. In this case, it describes the percentage of reserved days w.r.t. available days. Understanding which aspects influence the different levels of the OvR and the OcR is useful in the management of host Airbnb activity and improving results in time. Investigating the lowest and highest values of the OvR and the OcR makes it possible to understand the correct way to attract guests and make the activity profitable.

For this reason, we have tried to estimate the impact of specific elements on five different levels of the OvR and the OcR and, consequently, answer the following research question:

$$Q_1$$:
Which variables can influence different levels of the *overall rating* and the *occupancy rate*?

To answer this research question, the proportional odds model as defined in Yee (2015), among others, was used. This model identifies the elements that influence the probability of the response variables assuming a specific value. It provides relevant information for hosts to understand which elements can help with attaining specific OcR and OvR values. Consequently, hosts can understand which aspects are useful for improving their performance and achieving the highest results in terms of the OvR and the OcR. They can discover which covariates make it possible to move from one level to the next in a continuous effort to attain the best results.

The proportional odds model was specified taking into account two different groups of variables, the managerial variables and the characteristics of the accommodation. The managerial variables identify aspects of host activities that can be directly chosen by hosts (Contu et al. 2019). In fact, before appearing on the Airbnb platform, a host decides and declares specific aspects related to their activity, such as the minimum and maximum number of nights for reservations, how far in the future guests can book, cleaning fees, weekly discounts, and special prices and other aspects related to the activity management (https://www.airbnb.com/b/setup) (accessed on December 9th 2021). All these aspects are related to the services offered, additional fees that may apply, and how to build and conduct relationship with a guest. They are directly decided and managed by the host (Contu et al. 2019). The second group of variables identifies the *characteristics of the accommodation*, such as the accommodation type, size, and distance to the center of the city. They do not change quickly nor are they decided by hosts. However, if someone decides to invest in a house he owns and rent it on Airbnb, then it may be useful to know which aspects can influence the Airbnb results. The impact of these variables has been confirmed in the literature by Varma et al. (2016), which underlines how guests choose Airbnb accommodations for their *relatively more personal atmosphere than hotels* (Varma et al. 2016, p. 233), and by Guttentag et al. (2018), which identifies household amenities and the homely feel as important motivations for guests. Recently, Airbnb introduced an online section called *Airbnb plus stays*, where the most beautiful and charming accommodations are available for booking. This Airbnb section reinforces the necessity of considering aspects related to the accommodation in the attribution of the Superhost badge.

We assumed that the variables related to the services and type of place can have an impact that changes at different OvR and OcR levels.

We believe that the answer to the first research question can serve to define a framework that supports hosts. The results will offer the possibility of analyzing the capacity of the covariates to explain the different levels of the response variables. The results will suggest which variables can characterize a specific level of the OvR and the OcR and which variables can support moving from one level to the next.

The second objective focused on the characteristics of tourism destinations. Specifically, we aimed to understand if there are differences in variable impacts among the tourist cities. As stated already, the analysis focused on the cities of Rome, Milan, Venice, and Florence. The first two cities can be considered tourist cities, but also business cities. The others can be considered strictly tourist cities. We hypothesize to identify differences in variable impacts among these cities. Specifically, we aimed to answer the following research question:

- $Q_2$: Can differences in variable impacts among tourism destinations be identified?

To answer this second research question, we investigated the results obtained by applying the proportional odds model. It is important to identify differences among cities because we believe that each city presents specific characteristics and a different kind of tourism: some cities are only tourism cities, while others are also business cities. We hypothesized that guests search for different services in different destinations, thus each variable can have a different impact with respect to a specific OvR and OcR level and the analyzed destination.

In fact, answering the above two research questions made it possible to define a guide that supports hosts in their efforts to enhance their OvR and OcR levels. This guide contains the most significant aspects of the managerial variables and accommodation characteristics that impact on the OvR and the OcR. Moreover, based on differences between cities, the guide suggests which variables can support hosts concerning different territories.

Finally, the recent COVID-19 pandemic and its negative impact on Airbnb suggest the need to replicate the analysis in order to understand if there are differences in host performance between the pre-COVID-19 period and COVID-19 pandemic period. Different researchers have investigated the effect of the pandemic on Airbnb activities, underlining its disruptive impact (see, for instance, Dolnicar and Zare 2020; Jang and Kim 2022; Boros et al. 2020). We would have conducted the analysis for both response variables, but the data related to the OcR was not available, so we focused only on the OvR. In the literature, some researchers have investigated the impact of COVID-19 on the OcR (see, for instance, Ştiubea 2021; Boros et al. 2020; Llaneza Hesse and Raya Vílchez 2022), but not, to our knowledge, on the OvR. For this reason, we tried to answer the following research question:

- $Q_3$: Which variables can influence different levels of the overall rating during the COVID-19 pandemic? Can differences be recorded in the OvR between the pre-COVID-19 period and the COVID-19 pandemic period?

To answer this third research question, we replicated the proportional model on the OvR, taking into account the two different groups of variables and the four above-mentioned cities.

We focused on the 2021 data, the most recent (as at the time of this study) on the Inside Airbnb platform. We hypothesize to identify differences in variable impacts, particularly taking into account the characteristics of the accommodation.

To conclude, answering the third research question can guide hosts in their efforts to overcome the difficult situation generated by the pandemic. Moreover, it makes it possible to identify elements that impact on the positive perception of the Airbnb experience in difficult situations such as the COVID-19 pandemic.

### Data

The study was performed using two different datasets. The first was purchased from AirdDNA, a company that manages Airbnb data. We considered three groups of variables: *dependent variables* (that is the OvR and the OcR), *managerial variables* and the *characteristics of the accommodation*. Specifically, the managerial variables are the following:

- *Cancellation Policy*. Hosts can choose to apply three different kinds of cancellation policies: flexible, moderate, and strict. The cancellation policy aims to protect both guests and hosts alike. To introduce this variable in the model, it has been transformed into a binary variable. It assumes a value equal to 0 if the host chooses the flexible policy and a value equal to 1 if the choice is the strict or moderate policy.
- *Response Rate*. The percentage of time a host answers to potential guests within 24 hours.
- *Max Guests*. The maximum number of guests that can be hosted in the accommodation.
- *Response Time*. The amount of time that the guests must wait to obtain an answer to their questions.
- *Extra People Fee*. The price to pay to add one or more persons to the reservation.
- *Minimum Stay*. The minimum number of days that must be booked.
- *Security Deposit*. The payment (or not) of the deposit.
- *Business Ready*. The services that can support business travelers, such as Wi-Fi, a laptop-friendly workspace, iron, hangers, shampoo, hairdryer, and all the other essentials such as toilet paper and clean towels.
- *Instant book Enabled*. A service that allows guests to book accommodation without explicit host approval, facilitating the reservation process.
- *Superhost*. A binary variable that assumes a value equal to one when the host is a Superhost, otherwise, the value is equal to zero.
- *Number of Reviews*. The number of reviews published by guests.

The group of characteristics of the accommodation is composed of the following four variables:

- *Property Type*. Different types of accommodation can be rented on Airbnb. Specifically, it is possible to rent more than 25 different types of accommodation, like apartments, castles, boats, and caravans. The variable is included in the model and transformed into a binary variable. The value 1 is attributed to the accommodation type “apartment”, whereas the value 0 is attributed to all the other types.
- *Listing Type*. Three different listing types are offered on the Airbnb platform: the entire home, the private room, and the shared room. The variables have been transformed into binary variables. It is attributed to the value 1 when the entire accommodation is offered and the value 0 when a single room is rented.
- *Bedrooms*. The number of bedrooms in the accommodation.
- *Bathrooms*. The number of bathrooms in the accommodation.
- *Distance to the City Center*. For each accommodation has been calculated the distance from the center of the city. We identify the Pantheon as center of Rome city, *Piazza del Duomo* for the Milan, *Piazza San Marco* for Venice and *Piazza della Signoria* for Florence.

In order to use the dependent variables OcR or OvR into the model, they have been transformed into ordinal variables with five levels. OcR has been categorized by the following classes: *very low* $=[0,0.25]$, *low* $=(0.25,0.50]$, *medium* $=(0.50,0.75]$, *high* $=(0.75,0.90]$, *very high* $=(0.90,1.00]$. Instead, OvR has been categorized by the following classes: *very low* $=[0,2]$, *low* $=(2,3]$, *medium* $=(3,4]$, *high* $=(4,4.8]$, *very high* $=(4.8,5]$.

Airdna Data refers to 2016.

The second dataset was downloaded from the webpage of Inside Airbnb, a mission-driven project that provides data about Airbnb’s impact. The data was sourced from publicly available information on the Airbnb site (http://insideairbnb.com/). This dataset contains less information than the AirDNA dataset. Specifically, it does not contain the response variable OcR, nor the covariates *Business Ready*, *Extra People Fee*, *Security Deposit* and *Cancellation Policy*.

This second dataset refers to 2021. It was chosen to investigate the possible different levels of variable impact during the pre-COVID-19 and COVID-19 pandemic phases.

The geographical area considered in the analysis is composed of tourism cities. The four most visited cities in Italy were analyzed, namely Rome, Milan, Venice, and Florence. The choice of these cities lies in their importance as touristic destinations: most tourists visiting Italy stay in one of these cities.

According to the official tourism data of 2016 (the data related to the tourism flow in 2021 are yet to be published by the Italian National Institute of Statistics), the four most visited cities in Italy were Rome, with more than 29 million tourists, followed by Milan, Venice, and Florence (see Table 1).

**Fig. 1:**
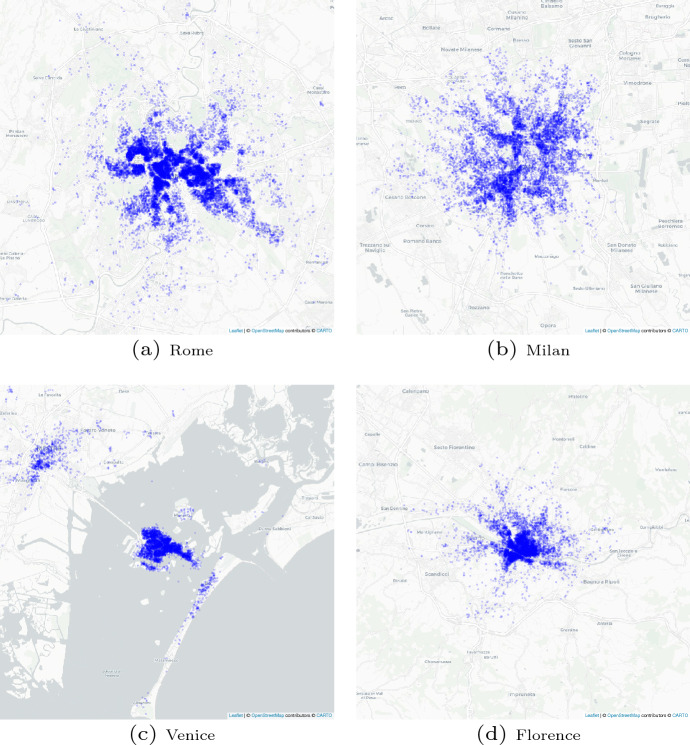
Airbnb accommodation distributions in the four cities analyzed, 2016

**Fig. 2:**
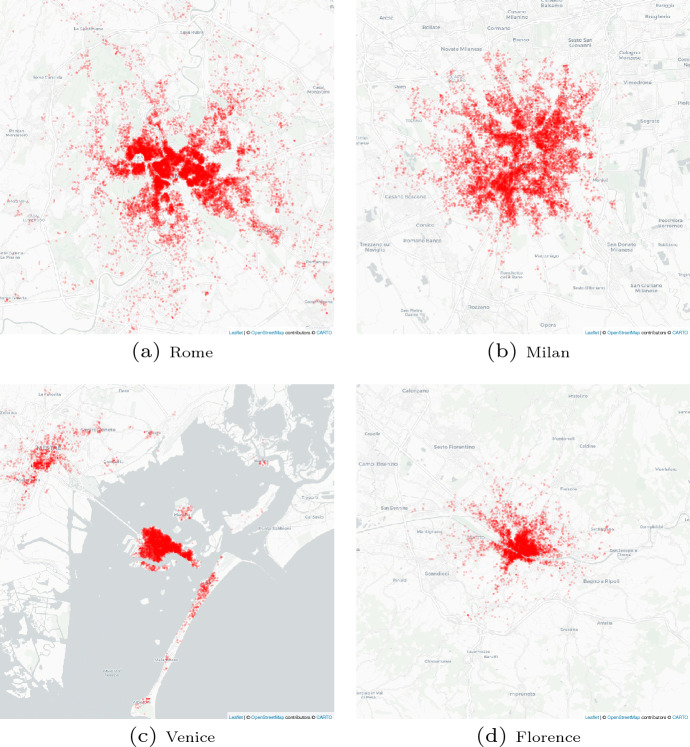
Airbnb accommodation distributions in the four cities analyzed, 2021

Analyzing the dataset related to 2016 and 2021, it was discovered that these four cities have the highest number of accommodations in Italy. This means that in Italy, Airbnb accommodations are located in the well-known and most visited tourist cities. Moreover, analyzing Figures 1 and 2, we observe how the Airbnb accommodations are located near the city center and tourist attractions. These findings are totally in line with the view expressed in Gutiérrez et al. (2017), which argues that Airbnb accommodations are located close to the main attractions; the results of Dudás et al. (2017), which confirm that Airbnb accommodations are concentrated in specific areas of tourist cities; and the observations of Romano (2021) and Celata and Romano (2022) that city centers of the most historical and tourist cities in Italy are occupied by Airbnb accommodations.

**Table 1:** Rank of tourism flow and Airbnb accommodation

Tourism official flow 2016			Airbnb accommodations 2016			Airbnb accommodations 2021		
Rank	City	Bed nights	Rank	City	Number	Rank	City	Number
1	Rome	25,191,580	1	Rome	19,042	1	Rome	24,626
2	Milan	10,976,244	2	Milan	11,057	2	Milan	16,785
3	Venice	10,511,788	3	Florence	77,533	3	Venice	7352
4	Florence	9,334,085	4	Venice	5625	4	Florence	6638

### Research method

The model used to carry out the analysis is the proportional odds model. It is applied when the response *Y* is ordinal variable and modeled in terms of the cumulative probabilities $P(Y \le j \mid x)$. Specifically. $Y$ is assumed to be a factor with ordered values $1,2,\dots ,J+1$.

The proportional odds model for a general ordinal *Y* can be written as (Yee 2015):

1 $$\begin{aligned} \text {logit} \left( P(y \le j \mid x)=\eta _{j}(x) \right) \end{aligned}$$

subject to the constraint

2 $$\begin{aligned} \eta _{j}(x)= \beta _{(j)1}^{*} +x^{T}_{[-1]} \beta ^{*}_{[-(1:M)]}, j=1, \dots , M \end{aligned}$$

with $j=1,\dots ,J$ where *j* identifies the level of *y* and moves from 1 to *J*, $M$ is the number of linear predictors $\eta _j$ and $M=J$ ; ${\mathbf {x}}_{[-1]}$ is the ${\mathbf {x}}$ with the first element deleted, $*$ denotes the regression coefficient that are to be estimated (Yee 2015, p. 11). In this analysis, we considered the probability of $P(Y \ge j)$, in specific:

3 $$\begin{aligned} \text {logit} \left( P(Y \ge j + 1\mid x) =\eta _{j}(x) \right) , \quad j= 1, \dots , M \end{aligned}$$

since $M = 1$ coincides with logistic regression. The model has been chosen to evaluate the impact that each variable can have on different levels of response variables. Moreover, it allows identifying which aspect can support moving from one level to another. Since the probability to $P(Y \ge j)$ is calculated, the results identify which elements can support the improvement of Airbnb’ performance.

Using the proportional odds models, we defined 16 different models to evaluate the relationship of the two dependent variables to the two groups of independent variables by city.

## Results and discussions

In this section, we describe the results obtained using the four models for each city. Firstly, we consider the models with the OcR as a response variable and then those with the OvR.

### OcR models

Analyzing the results from Tables 2, 3, 4, and 5 we found that the managerial variables and the characteristics of the accommodation were, in most cases, statistically significant ($p < 0.05$). The impact of the variables generally decreased with increasing values of the OcR, except for the variable *Instant book Enabled* whose impact increased. Moreover, the positive and negative impacts remained steady across all categories. Some differences in terms of positive and negative impacts were noted between cities.

Analyzing the impact of each variable in greater detail, we observed, firstly, that the variable *Instant book Enabled* exerted a positive impact on the OcR. Gunter and Önder (2018) and Benítez-Aurioles (2018) found that this positive impact was related to the preference of guests for hotel-like booking processes. This impact might also be related to a sense of greater freedom that guests look out for while planning their holidays. In fact, Airbnb accommodations have fewer constraints and schedules than hotels, and this feeling of freedom and ease gets stronger when one does not need to wait for one’s host’s confirmation. The result suggests that improving the *Instant book Enabled* is fundamental to operating as a hotel and allowing more freedom in the Airbnb experience.

Secondly, a significant impact of the variables *Response Time* and *Response Rate* was observed. Generally, the *Response Rate* is perceived as a signal of reliability and honesty and is crucial to building a trustworthy relationship between hosts and guests from the very beginning (Xie and Mao 2017). It can be considered as a measure of host responsiveness and is one of the variables that influence Airbnb demand (Gunter and Önder 2018). Our results suggest that communication, when required, has to be quick and immediate.

Thirdly, we observed a significant impact of the variable *Cancellation Policy* on the OcR in Rome and Milan. In general, this variable defines the terms and conditions of cancelling a reservation (Benítez-Aurioles 2018). The results suggest that a strict cancellation policy influences the different OcR levels. In fact, such a policy is a guarantee that a host will lose reservations and money. Benítez-Aurioles (2018) demonstrated that hosts who managed their accommodations as professionals used the same strategy as hotels and chose specific cancellation policies. A clear *Cancellation Policy* can guarantee guests, because it is another signal of reliability and honesty. Consequently, we can argue that the presence of cancellation policies is an indication of professional management; it is interpreted here as a sign that a host treats their activity as a business and wants to project a reliable image to potential guests. An insignificant impact was observed for the cities of Florence and Venice. This difference in impacts between the two groups of cities can be justified by hypothesizing that there was a major presence of business tourists in Rome and Milan. This kind of tourists needs a major guarantee during their travel, compared with tourists that spend their vacation in tourism and cultural destinations.

Moreover, a positive impact on the response variable OcR was noted for the variable *Superhost*. The result is in line with the literature. In fact, Xie and Mao (2017) showed the important impact of attaining a Superhost status on the occupancy rate. The authors state that *having more reservations in the subsequent month depends in part on becoming a Superhost* (Xie and Mao 2017, p. 16). In fact, the Superhost badge is a guarantee for guests, who normally associate the badge with professionality; this sense of professionality mitigates the perceived risk of staying in the private house of a stranger (Xie and Mao 2017). We can state that the Superhost badge influences the OcR because it suggests a positive image of the host.

This positive impact of a host’s image is also confirmed by the positive and increasing impact of the variable *Number of Reviews*. This variable had a common positive effect for all destinations. The relevant impact of this variable was demonstrated in Qiu et al. (2018), which found that the number of reviews had a stronger effect than the average rating. This means that if the number of reviews increased, the probability of obtaining a better result in terms of the OcR increased.

The results observed for the variable *Extra People Fee* are particularly interesting. In most cases, the probability of obtaining the highest OcR classes was negatively influenced by this variable. These results suggest that to reach high OcR values, it is necessary to not ask for more money from guests. This result is confirmed by the negative and significant impact observed for the variable *Security Deposit*. We can state that these fees are not positively perceived by guests and they impact negatively on the OcR.

Moreover, it has been recorded as a negative influence for the variable *Max Guests*. This influence increased from the lowest to the highest OcR levels. This result suggests that, generally speaking, guests prefer smaller accommodations. This statement seems confirmed by the significant and negative impact of the variables *Bedrooms* and *Bathrooms* on the probability of attaining a high OcR level. The results also suggest that an increase in the size of an accommodation has no positive impact on the rate of occupied days of Airbnb accommodations in the studied Italian cities.

Additionally, considering the other characteristics of accommodations, we found that the *Property Type* had a positive and, in most cases, statistically significant impact on the different OcR levels. The result highlights how renting an apartment, instead of a room, improves the OcR in all cities. This result is in line with the findings of previous studies that guests chose Airbnb accommodations for the *relatively more personal atmosphere than hotels* (Varma et al. 2016, p. 233), the major independence with respect to traditional hotels (Flognfeldt and Tjørve 2013), and the homely feel and presence of household amenities (Guttentag et al. 2018).

This result is confirmed also by the positive impact of the variable *Listing Type* on Rome and Milan. Renting an entire accommodation improved the possibility of increasing the OcR for these cities. Also, for these cities, we believe that this impact was affected by business tourists who need more privacy and space to work, which are two of the factors that determined this positive impact. In fact, Airbnb recently developed and promotes a new service, *Airbnb for work*. This service offers a selection of houses with helpful tools for working and identifying available accommodation for delegates near the event location, and assist with the organization of group trips (von Briel et al. 2021). This new project underlines how business tourism is relevant to Airbnb and the necessity of delivering adequate services that meet the requirements of business tourists. On the other hand, a negative impact of the *Listing Type* was noted for the highest OcR values for Florence and Venice, confirming the major willingness of leisure and cultural tourists to share spaces. This willingness is justified by the desire to meet new people and save money during trips.

Finally, a negative and significant impact of the variable *Distance to the City Center* on the OcR was observed. This impact increased with an increasing OcR. The result suggests that the proximity of an accommodation to the city center increases the possibility of renting the accommodation for more days and improves host performance. Different researchers have underlined how the position of an Airbnb accommodation in general (see, for instance, Varma et al. 2016; Ioannides et al. 2019; Rubino et al. 2020) and the reduced distance to the center of the city in particular (see, for instance, Gunter and Önder 2018) can influence the choice of guests. Moreover, Gutiérrez et al. (2017) and Quattrone et al. (2016, 2018) have emphasized the major presence of Airbnb accommodations in city centers, and Quattrone et al. (2016) has shown how the demand for Airbnb accommodations is concentrated closer to city centers. Consequently, we can state that city centers represent the best meeting point of Airbnb demand and supply.

These findings suggest that the OcR depends not only on the available services but also on the characteristics of the accommodation. This result is extremely relevant in the study of Airbnb.

**Table 2:** Proportional model applied on Milan

	$\text {logit}(P[Y\ge { low}])$	$\text {logit}(P[Y\ge { medium}]])$	$\text {logit}(P[Y\ge { high}]])$	$\text {logit}(P[Y\ge { very high}]])$
Milan				
*Occupancy rate (OcR)*				
(Intercept)	−1.114***	−1.644***	−2.661***	−3.638***
Max Guests	−0.045***	−0.073***	−0.088***	−0.069*
Response time	0.000***	0.000***	0.000***	0.000
Security Deposit	−0.065***	−0.097***	−0.032***	0.070
Extra People Fee	0.121***	0.016***	−0.190***	−0.412***
Business Ready	−0.067***	−0.089***	−0.077***	−0.189
Instant book Enabled	0.641***	0.848***	1.143***	0.679***
Number of Reviews	0.015***	0.022***	0.031***	0.031***
Response Rate	0.008***	0.004***	0.001***	−0.002
Superhost	0.621***	0.372***	0.009***	−0.283*
Cancellation Policy	0.145***	0.017***	−0.042***	0.033
(Intercept)	0.193*	−0.450***	−1.518***	−3.071***
Distance center	−0.032*	−0.041**	−0.057**	−0.009
Listing Type	0.310***	0.297***	0.276***	0.199
Property Type	0.149*	0.066	0.136	0.055
Bedrooms	−0.197**	−0.239***	−0.270***	−0.222*
Bathrooms	−0.112*	−0.261***	−0.369***	−0.259
*Overall rating (OvR)*				
(Intercept)	0.286***	1.789***	0.565***	−0.836***
Max Guests	−0.061***	−0.082***	−0.155***	−0.061***
Response Time	0.000***	0.000***	0.000	0.000**
Security Deposit	0.250***	0.315***	0.282***	0.174***
Extra People Fee	0.410***	0.112***	0.225***	−0.170***
Business Ready	−0.325***	−0.115***	0.155	0.081
Instant book Enabled	−0.836***	−0.672***	−0.600***	−0.241***
Number of Reviews	0.015***	0.016***	0.010***	−0.014***
Response Rate	0.051***	0.021***	0.012***	0.001
Superhost	0.432***	0.508***	0.851***	1.567***
Cancellation Policy	−0.271***	0.022***	0.123*	−0.110*
(Intercept)	4.434***	3.434***	1.682***	−1.300***
Distance center	0.011	0.072	0.018	0.001
Listing Type	0.229	0.098	−0.015	−0.131*
Property Type	0.475	0.409*	0.254**	0.145*
Bedrooms	0.175	0.016	−0.037	−0.054
Bathrooms	−0.009	−0.128	−0.071	0.140**

**Table 3:** Proportional model applied on Rome

	$\text {logit}(P[Y\ge { low}])$	$\text {logit}(P[Y\ge { medium}]])$	$\text {logit}(P[Y\ge { high}]])$	$\text {logit}(P[Y\ge { very high}]])$
Rome				
*Occupancy rate (OcR)*				
(Intercept)	−0.957***	−1.697***	−2.846***	−4.481***
Max Guests	−0.036***	−0.035***	−0.047***	−0.043***
Response Time	−0.001***	−0.001***	0.000***	0.000***
Security Deposit	−0.113***	−0.168***	−0.264***	−0.369***
Extra People Fee	0.037***	−0.055***	−0.163***	−0.239***
Business Ready	0.209***	0.245***	0.319***	0.377***
Instant book Enabled	0.486***	0.485***	0.503***	0.455***
Number of Reviews	0.017***	0.022***	0.031***	0.040***
Response Rate	0.010***	0.006***	0.002***	0.000***
Superhost	0.548***	0.463***	0.366***	0.303***
Cancellation Policy	0.122***	0.103***	0.099***	−0.042***
(Intercept)	0.810***	−0.018	−1.102***	−2.826***
Distance center	−0.081***	−0.109***	−0.124***	−0.108***
Listing Type	0.286***	0.284***	0.135*	−0.014
Property Type	0.326***	0.249***	0.272***	0.135
Bedrooms	−0.072**	−0.112***	−0.228***	−0.157*
Bathrooms	−0.216***	−0.231***	−0.302***	−0.300**
*Overall rating (OvR)*				
(Intercept)	0.872***	0.920***	−0.403***	−0.858***
Max Guests	−0.133***	−0.119***	−0.056***	−0.010***
Response Time	−0.001***	0.000***	0.000***	0.000**
Security Deposit	0.391***	0.182*	0.289***	0.031
Extra People Fee	0.806***	0.638***	0.290***	−0.058
Business Ready	0.348***	0.370***	0.325***	0.128
Instant book Enabled	−0.181***	−0.223***	−0.109***	−0.190***
Number of Reviews	0.015***	0.016***	0.012***	−0.014***
Response Rate	0.033***	0.023***	0.016***	−0.002*
Superhost	0.646*	0.702***	0.934***	2.123***
Cancellation Policy	0.558***	0.304***	0.164***	−0.087***
(Intercept)	4.386***	3.504***	1.455***	−1.453***
Distance center	0.135**	0.055*	0.013	0.030***
Listing Type	0.656**	0.279*	0.307***	−0.171***
Property Type	0.412	0.321*	0.236***	−0.179***
Bedrooms	−0.085	−0.012	0.022	0.068*
Bathrooms	−0.211	−0.186*	−0.115**	0.125***

**Table 4:** Proportional model applied on Florence

	$\text {logit}(P[Y\ge { low}])$	$\text {logit}(P[Y\ge { medium}]])$	$\text {logit}(P[Y\ge { high}]])$	$\text {logit}(P[Y\ge { very high}]])$
Florence				
*Occupancy rate (OcR)*				
(Intercept)	−0.889**	−1.707***	−2.470***	−3.345***
Max Guests	−0.068***	−0.075***	−0.108***	−0.105**
Response Time	0.000***	0.000***	0.000*	0.000
Security Deposit	−0.167***	−0.129***	−0.072**	0.093
Extra People Fee	0.307***	0.089***	0.052***	−0.120
Business Ready	0.045	−0.050	0.309***	0.034
Instant book Enabled	0.419***	0.371***	0.253***	0.003
Number of Reviews	0.014***	0.018***	0.023***	0.026***
Response Rate	0.011***	0.010**	0.003	−0.005
Superhost	0.561***	0.414***	0.175***	0.024
Cancellation Policy	−0.003	−0.045	−0.089	−0.037
(Intercept)	1.005***	0.261**	−0.697***	−2.224***
Distance center	−0.075***	−0.121***	−0.191***	−0.190**
Listing Type	0.009	−0.067	−0.313***	−0.470**
Property Type	0.383***	0.276***	0.198*	0.125
Bedrooms	−0.124**	−0.220***	−0.340***	−0.302**
Bathrooms	−0.198***	−0.187***	−0.170*	−0.205
Overall rating (OvR)				
(Intercept)	−0.373	−0.848***	−0.641***	−1.079***
Max Guests	−0.115**	−0.086***	−0.029***	0.024***
Response Time	−0.001**	−0.001***	0.000***	0.000
Security Deposit	0.287	−0.148***	0.081***	−0.041
Extra People Fee	0.910***	0.933***	0.358***	−0.043
Business Ready	0.482	0.270***	0.182***	0.162
Instant book Enabled	−0.066	−0.167***	−0.191***	−0.311***
Number of Reviews	0.012*	0.012***	0.008***	−0.011***
Response Rate	0.050***	0.044***	0.021***	−0.002
Superhost	0.712	0.728**	0.997***	2.014***
Cancellation Policy	0.259	0.032***	0.021***	0.030***
(Intercept)	4.048***	2.795***	1.410***	−1.727***
Distance center	0.192***	0.198***	0.052	0.051*
Listing Type	0.604***	0.061***	0.200*	0.136
Property Type	1.492***	1.248***	0.442***	−0.161
Bedrooms	−0.372***	−0.241***	−0.075	−0.019
Bathrooms	−0.140***	0.145***	0.024	0.292***

**Table 5:** Proportional model applied on Venice

	$\text {logit}(P[Y\ge { low}])$	$\text {logit}(P[Y\ge { medium}]])$	$\text {logit}(P[Y\ge { high}]])$	$\text {logit}(P[Y\ge { very high}]])$
Venice				
*Occupancy rate (OcR)*				
(Intercept)	−0.020	−0.311***	−2.047***	−3.841***
Max Guests	−0.108***	−0.094***	−0.101***	−0.106**
Response Time	0.000***	0.000***	0.000*	−0.001
Security Deposit	−0.099**	−0.142***	−0.361***	−0.579***
Extra People Fee	0.320***	0.072***	−0.071***	−0.238
Business Ready	0.092	0.061	−0.236	0.086
Instant book Enabled	0.149***	0.167***	0.235***	0.012
Number of Reviews	0.014***	0.018***	0.024***	0.027***
Response Rate	0.006***	−0.003***	−0.003***	0.000
Superhost	0.505***	0.606***	0.439***	0.088
Cancellation Policy	−0.149	−0.264***	−0.308**	−0.059
(Intercept)	1.474***	0.499***	−1.122***	−2.844***
Distance center	−0.038***	−0.040***	−0.070***	−0.081*
Listing Type	−0.169	−0.122	−0.341**	−0.438
Property Type	0.161	−0.010	0.290*	0.323
Bedrooms	−0.111*	−0.127**	−0.231**	−0.238
Bathrooms	−0.314***	−0.432***	−0.410***	−0.245
*Overall rating (OvR)*				
(Intercept)	5.256*	1.998**	−0.356	−1.408**
Max Guests	−0.008	−0.011	−0.029	−0.008
Response Time	0.001	0.000	0.000	0.000
Security Deposit	0.354	0.706***	0.440***	0.015
Extra People Fee	−0.467	0.004	0.305***	−0.079
Business Ready	−0.125	−0.495	−0.269	0.137
Instant book Enabled	0.041	−0.172	−0.573***	−0.117
Number of Reviews	0.075***	0.053***	0.018***	−0.017***
Response Rate	−0.017	0.005	0.013***	0.000
Superhost	2.665	2.980**	3.577***	2.246***
Cancellation Policy	0.298	−0.136	−0.019	−0.115
(Intercept)	4.942***	2.806***	1.257***	−1.714***
Distance center	−0.001	0.041	0.001	−0.009
Listing Type	0.837	0.541*	0.558***	0.210
Property Type	−0.374	0.108	−0.255*	−0.339**
Bedrooms	−0.226	−0.041	0.049	0.025
Bathrooms	0.147	0.164	−0.055	0.147*

### Overall rating

We also investigated the variables that affected the OvR in the two different phases: the pre-COVID-19 and COVID-19 pandemic phases.

#### Overall rating pre Covid 19 pandemic

We first analyzed the pre-pandemic results. Specifically, analyzing the results from Tables 2, 3, 4, and 5 we observed that the covariates were not always statistically significant. We observed a significant impact of the managerial variables on the different OvR levels for the cities of Rome and Milan, on the mid OvR levels for Florence, and on the fourth OvR level for Venice. Generally, the managerial variables could not explain the majority of the OvR levels for Venice. The unique variable that had an impact on the different OvR levels for Venice was the *Number of Reviews*.

The impact of the variables generally decreased with increasing OvR levels, except for the variable *Superhost* whose impact increased with an increasing OvR. An OvR greater than 4.8 is one of the aspects that conditions the attribution of the Superhost badge. Moreover, Gunter (2018) has demonstrated that this is the aspect with the highest marginal effect on the probability of becoming a Superhost. Consequently, we can state that a high OvR and attaining a Superhost status are linked and one conditions the other.

We also observed how a positive or negative impact of the covariates remained mainly steady across all levels. For instance, the variable *Cancellation Policy*, which was statistically significant for the different OvR levels for the city of Milan, exerted a negative impact on the lowest and on the highest levels and a positive impact on the mid levels. However, we know that the presence of a cancellation policy guarantees more guests because it is an indication of a host’s reliability and honesty. We believe that when a guest has a terrible experience, a strict cancellation policy can be perceived as a further limitation and negative aspect of the vacation. Consequently, it impacts negatively on the general judgment expressed at the end of the Airbnb experience. Similarly, when a guest has a very positive experience, restrictions on cancellation can be perceived as a disturbing element that might have a negative impact on the judgment of the experience. Similar results were obtained for the city of Rome, where a contrasting and negative impact was observed for the highest OvR level.

Analyzing the impact of each variable, we noted how, for instance, the variable *Instant book Enabled* had a negative impact on the variable OvR for Rome, Milan, and Florence, and the fourth class for Venice. An opposite trend was observed with respect to the OcR. This difference may be justified if we consider that although guests judge positively this kind of service when they have to book accommodation (instant book offers freedom and speed in the booking phase), the reduced contact with hosts at the same time impacts negatively on the general judgment relative to the experience. In fact, the rental of an Airbnb accommodation is based on the relationship between the guest and the host (Tussyadiah and Park 2018). For this reason, we can state that the possibility of speaking and meeting with the host can generate a positive feeling and positively influence the perception of the Airbnb experience. For this reason, a host should promote this service to facilitate the booking experience of guests, but at the same time attempt to create a positive and supportive relationship with guests.

In addition, a positive and significant impact of the variable *Business Ready*on the OvR was observed for the cities of Rome (from the second to the fourth classes) and Florence (from the third to the fourth classes). This positive impact shows how guest judgments are related to the quality and quantity of the supplied services. The positive impact suggests that guests like to receive the same services that hotels normally offer. The results are confirmed by the findings of previous studies that *Airbnb users’ are primarily attracted to the service by its practical advantages* (Guttentag et al. 2018, p. 354) and that *hosts should be aware of the fact that guests request a specific standard, besides the interactive part of the hosting experience* (Lalicic and Weismayer 2018, p. 89). However, the variable *Business Ready* was not significant for Venice and for the last two classes of Milan. The other classes present a negative impact on Venice. The result suggests that for these two cities the business services are not relevant, but other aspects related more to the host image influence the judgment of the Airbnb experience. For instance, for Milan it is crucial that an accommodation is managed by a Superhost, and for Venice it is important that a large number of reviews guarantees the reliability of the host.

A significant impact was also recorded for some variables related to the characteristics of the accommodation. For instance, in most cases, the variable *Listing Type* positively influenced the OvR for hosts in the different cities. The findings demonstrate that renting an entire accommodation improves the probability of obtaining better reviews. The result suggests that judgment relative to the Airbnb experience depends not only on the available services but also on the characteristics of the accommodation. This novel result is extremely relevant to the study of Airbnb.

Taking into account the accommodation size, it is interesting to underline how *Bathrooms* significantly and positively impacted on the last OvR classes. This means that the presence of an elevated number of bathrooms improves the experience and boosts the OvR scores. On the contrary, this variable impacted negatively on low OvR values.

The results related to the variable *Distance to the City Center* are very interesting. This variable was not significant for the cities of Milan and Venice, but it had a positive impact for the cities of Rome and Florence. This means that the highest OvRs are obtained when accommodations are far away from the city center.

In the literature, different researchers have investigated the impact the location of an accommodation can have on guest satisfaction. For some researchers, location is one of the most critical aspects that influence the OvR (see, for instance, Tussyadiah and Zach 2017; Luo and Tang 2019). For others, location does not influence guest satisfaction (see, for instance, Sthapit and Jiménez-Barreto 2019).

In the current study, proximity to the city center was not significant for Venice, probably because the city is quite small and the entire city can be perceived as a center. On the contrary, the city of Milan is very big and other aspects related to location impacted more on the OvR. As demonstrated by Luo and Tang (2019), proximity to specific points of interest and transportation convenience may have greater impact on the OvR than staying near the city center.

Moreover, the positive impact for the cities of Rome and Florence may be explained by the limited access to public transportation in the centers of these cities. In fact, some central neighborhoods in Rome and Florence are pedestrian areas, so reaching a specific destination far away from the city center can be difficult. We believe that the impact of this variable on the OvR was conditioned by the characteristics and size of the city.

To sum up, we can state that these results underline another time how the judgment expressed by the guest is influenced by the size of the accommodation.

#### Overall rating during Covid 19 pandemic

We also tried to understand if the impact of the managerial variables and the characteristics of the accommodation remained the same during the COVID-19 pandemic. Specifically, we evaluated the impact of these variables on the OvR in 2021.

Analyzing Table 6, we discovered a significant change in the variable impact before and during the COVID-19 pandemic. Firstly, all managerial variables became significant and impacted on the OvR during the pandemic. For instance, the variable *Superhost* became relevant for all levels of the OvR for all cities. This result underlines the desire of guests to spend their vacation in accommodations managed in a professional way. Guests preferred to deal with professional hosts who observed the COVID-19 restrictions and offered a safe stay. The results are confirmed by different studies that have underlined how only professional hosts were able to maintain fairly high performance during the pandemic (see, for instance, Dolnicar and Zare 2020; Garha and Azevedo 2022).

A significant change in impact between the two periods was found for the variables *Distance to the City Center*. This impact changed the sign and became relevant for all cities. The result suggests that proximity to the city center increased the possibility of reaching a high overall rating during the pandemic.

This is justified by the desire to reduce the use of public means of transport and physical contact with people.

Moreover, the number of bedrooms and bathrooms in an accommodation became one the most relevant aspects. The OvR was influenced by the size of the accommodation: a reduced number of rooms was preferred. This result is in line with a previous finding that *minimizing contacts between people may lead to benefits that are both egoistic in nature (namely, protecting oneself) and altruistic or social (as protecting others)* (Bresciani et al. 2021, p. 2).

The analysis related to the COVID-19 pandemic period seems to design an increase in similarities among variable impacts for the four cities. The managerial variables, distance to the city center, and size of the accommodation were aspects that influenced the OvR during the COVID-19 pandemic.

**Table 6:** Proportional model applied on 2021

	$\text {logit}(P[Y\ge { low}])$	$\text {logit}(P[Y\ge { medium}])$	$\text {logit}(P[Y\ge { high}]])$	$\text {logit}(P[Y\ge { very high}]])$
*Milan*				
(Intercept)	3.269***	2.956***	1.017***	−0.714**
Max Guests	−0.035***	−0.049***	−0.056***	−0.066***
Response Time	−0.001***	0.000***	0.000***	0.000***
Instant book Enabled	−0.397***	−0.730***	−0.778***	−0.469***
Number of Reviews	0.005***	0.006***	0.006***	−0.002***
Response Rate	0.011***	0.008***	0.013***	0.008***
Superhost	0.889***	0.994***	1.057***	1.383***
(Intercept)	3.479***	3.262***	2.197***	0.028
Distance center	−0.075***	−0.072***	−0.046**	0.013
Listing Type	1.408	0.123	−0.132	−0.389
Property Type	−0.868	0.182	0.238	0.222
Bedrooms	−0.190*	−0.326***	−0.149*	−0.184***
Bathrooms	0.153***	0.317***	0.191*	0.264***
*Rome*				
(Intercept)	−1.234***	−1.319***	−1.022***	−1.242***
Max Guests	0.066***	0.074***	0.037***	0.010***
Response Time	−0.001***	0.000***	0.000***	0.000***
Instant book Enabled	−0.111***	−0.266***	−0.110***	−0.245***
Number of Reviews	0.006***	0.006***	0.007***	−0.003***
Response Rate	0.055***	0.049***	0.031***	0.009***
Superhost	0.473***	0.618***	0.830***	1.438***
(Intercept)	3.804***	3.373***	2.099***	−0.434***
Distance center	−0.061***	−0.052***	−0.018**	0.025***
Listing Type	1.742	1.136	−0.234	0.072
Property Type	−0.736	−0.118	1.114***	0.148
Bedrooms	−0.050	−0.076	−0.068	−0.084**
Bathrooms	−0.055	−0.046	0.021	0.177***
*Florence*				
(Intercept)	−1.550***	−0.074***	−0.636***	−2.021***
Max Guests	0.063***	0.064***	0.032***	0.018
Response Time	0.000***	0.000***	0.001***	0.001***
Instant book Enabled	0.224***	−0.260***	−0.181***	−0.248***
Number of Reviews	0.005***	0.006***	0.006***	−0.002***
Response Rate	0.057***	0.034***	0.026***	0.016***
Superhost	0.882***	1.150***	1.277***	1.506***
(Intercept)	4.872***	3.895***	2.297***	−0.524***
Distance center	−0.270***	−0.035***	0.082	0.113***
Listing Type	1.292	1.341	1.378	0.067
Property Type	−1.194	−1.009	−0.857	0.242
Bedrooms	0.119***	0.113***	−0.083	−0.187***
Bathrooms	0.122***	−0.095***	0.128	0.315***
*Venice*				
(Intercept)	−1.449***	1.230***	−2.597***	−1.595***
Max Guests	0.179***	−0.006***	0.033***	0.002***
Response Time	−0.001***	−0.002***	0.001***	0.000*
Instant book Enabled	−0.091***	−0.565***	−0.243***	−0.223***
Number of Reviews	0.006***	0.006***	0.006***	−0.002***
Response Rate	0.057***	0.032***	0.047***	0.010**
Superhost	0.768***	1.037***	1.161***	1.822***
(Intercept)	4.579***	4.006***	2.396***	−0.639***
Distance center	−0.065	−0.063*	−0.043**	0.001
Listing Type	11.637	11.846	−0.568	−0.046
Property Type	−10.777	−10.736	1.748*	0.567
Bedrooms	−0.076	−0.254*	−0.208**	−0.090*
Bathrooms	0.160	0.179	0.090	0.229***

### Differences among cities

The results highlighted similarities and differences between cities. Analyzing the results concerning the OcR, we observed that, in general, the managerial variables and the characteristics of the accommodation impacted in a similar way for the different cities. However, some differences were noted in the last OcR levels for Milan, Florence, and Venice. The number of significant variables for these cities reduced. For instance, the highest OcR level for Florence was explained only by the variable *Number of Reviews*; whereas for Venice it was explained by the *Number of Reviews*,*Max Guests* and *Security Deposit*.

Moreover, contrasting impacts were found for some variables related to the characteristics of the accommodation. For instance, renting an entire accommodation in Rome impacted positively on the OcR. On the contrary, it presented a negative impact in Venice, where accommodation sharing was preferred to renting an entire apartment. This contraposition suggests the difference between the needs of guests who spend their vacation in Rome and those who do in Venice. Similar results were found in the analysis of aspects impacted the OvR. In this case, the managerial variables presented similar impacts for the cities of Rome, Milan, and Florence, whereas they only affected the fourth OvR level for Venice. A totally different impact was noted for the characteristics of the accommodation. These variables impacted the OvR for Florence and Rome, but not for Venice and Milan. In other words, only the managerial variables could explain the judgment of the entire Airbnb experience in Milan, and the accommodation type and size did not influence the review. For Venice, the *Number of Reviews* and other variables influenced the judgment of the Airbnb experience.

Particularly interesting are the results obtained for the variable *Distance to the City Center*. The variable presented different levels of impact for the cities in the period prior to the COVID-19 pandemic, but assumed a negative impact for all cities during the pandemic. This result suggests how the judgment related to the Airbnb experience changes with the needs of guests.

In general, to boost their OcR and OvR values, hosts should consider different elements that can influence their performance, depending on the cities where they operate, and evaluate differences in influence related to the pre-COVID-19, COVID-19 and post-COVID-19 pandemic period.

## Concluding remarks

Few researchers have investigated elements that are crucial to improving host performance on Airbnb. Our study aimed to identify elements that can significantly influence host activity. Some innovative aspects were explored in our analysis. Firstly, this is one of the few studies to use VGAM as defined by Yee (2015) for tourist analysis and, specifically, to investigate components that can influence the performance of tourism accommodations. We used a complex model, namely the proportional odds model, to investigate the impact of specific variables on different levels of the OcR and the OvR. Secondly, we investigated the impacts on the OcR and the OvR of two different kinds of variables: the managerial variables and the characteristics of the accommodation.

Moreover, the models were defined and the Airbnb data were applied such that the similarities and differences between cities could be identified. In fact, four models (two for the OcR and two for the OvR) were defined for four cities to investigate the impact of the different groups of covariates on the two response variables for these cities. The results made it possible to define a statistical framework that could explain host activity by taking into account the different tourism destinations.

We have seen that such variables as *Business Ready*, *Response Time*, *Number of Reviews*, and *Superhost* positively influence host performance. This means that hosts should consider improving these aspects to successfully manage their accommodations. The of these different elements makes it possible to create an enticing tourism offer and boost performance. Moreover, the provision of high-quality services and continuous support for guests ensures the attribution of the label *professional* to hosts. Since Airbnb accommodations seem to be becoming a substitute for the hotels, hosts should offer the same services as hotels but in a nicer and more informal environment. Hosts should serve as tourist managers offering high-quality and diversified services and a nice place to spend pleasant vacations. Professional management ensures hosts could maintain profitable Airbnb activities in the long run.

We discovered that the characteristics of the accommodation are fundamental to obtaining high results in terms of the OvR and the OcR. Renting entire apartments, when available, boosts the probability of improving host results. Even the number of bedrooms and bathrooms can influence the OcR and the general judgment of the guests. Hosts cannot easily change their accommodations, but they can in time modify them to meet the needs of guests.

Additionally, we discovered that the *Distance to the City Center* and size of the accommodation, measured through the variables *Bedrooms* and *Bathrooms*, became relevant during the COVID-19 pandemic, underling how the observance of social distancing was fundamental to a feeling of safety and a positive Airbnb experience. Our findings suggest that hosts should consider the characteristics of tourism destinations in the management of their accommodations. The level of development and the characteristics of the destination affect host activity and the variables that impact host performance and obtaining the Superhost badge.

In fact, our findings define a guide for hosts to improve their performance, since they suggest the different aspects that hosts should concentrate on. This study, of course, has some limitations. The first is related to the data sources. Airbnb does not share its data for analysis. For this reason, the research was realized using mostly scraped data. This kind of data presents some problems. The first problem is related to the technical activity involved in scraping data. The second problem involves the legal aspect, such as illegal access and use of data, breach of contract, and copyright issues. The last problem concerns possible ethical consequences in relation to the use of web data (Krotov et al. 2020). These problems notwithstanding, data scraping seemed to be the only possible way to obtain data for analysis. Moreover, we believe that web scraping can be considered as an inexpensive and easily accessible way of obtaining data to study phenomena that cannot otherwise be investigated.

The second limitation is a geographical limitation. The study has focused on the most visited Italian cities. It is not possible to know whether the results obtained for the cities apply to smaller or less touristic cities; or whether the location of a city influences the Airbnb activities of hosts in the city. For instance, it would be interesting to understand if cities located by the sea behave differently than cities located far from the sea.

Finally, additional research including more cities should be undertaken to see if the findings of this study apply to other territories. Cities in other countries should be analyzed to see if important differences would emerge: for instance, it would be very interesting to study host activity for different European capitals. Besides, the variables that influence rental activities should be analyzed for different tourist destinations.
